# Mycotoxin Regulatory Status in Africa: A Decade of Weak Institutional Efforts

**DOI:** 10.3390/toxins14070442

**Published:** 2022-06-29

**Authors:** Cynthia Adaku Chilaka, Jude Ejikeme Obidiegwu, Augusta Chinenye Chilaka, Olusegun Oladimeji Atanda, Angela Mally

**Affiliations:** 1 Institute of Pharmacology and Toxicology, Julius Maximilian University of Würzburg, Versbacher Str. 9, 97078 Würzburg, Germany; mally@toxi.uni-wuerzburg.de; 2 National Root Crops Research Institute, Umudike, Km 8 Umuahia-Ikot Ekpene Road, Umuahia P.M.B. 7006, Abia State, Nigeria; ejikeobi@yahoo.com; 3 Department of Nutrition and Forage Science, Michael Okpara University of Agriculture, Umuahia P.M.B. 7267, Abia State, Nigeria; chinenyeaugusta2016@gmail.com; 4 Department of Natural Sciences, Precious Cornerstone University, Ibadan 200235, Oyo State, Nigeria; olusegunatanda@yahoo.co.uk

**Keywords:** fungi, mycotoxin, legislation, food safety, food security

## Abstract

Food safety problems are a major hindrance to achieving food security, trade, and healthy living in Africa. Fungi and their secondary metabolites, known as mycotoxins, represent an important concern in this regard. Attempts such as agricultural, storage, and processing practices, and creation of awareness to tackle the menace of fungi and mycotoxins have yielded measurable outcomes especially in developed countries, where there are comprehensive mycotoxin legislations and enforcement schemes. Conversely, most African countries do not have mycotoxin regulatory limits and even when available, are only applied for international trade. Factors such as food insecurity, public ignorance, climate change, poor infrastructure, poor research funding, incorrect prioritization of resources, and nonchalant attitudes that exist among governmental organisations and other stakeholders further complicate the situation. In the present review, we discuss the status of mycotoxin regulation in Africa, with emphasis on the impact of weak mycotoxin legislations and enforcement on African trade, agriculture, and health. Furthermore, we discuss the factors limiting the establishment and control of mycotoxins in the region.

## 1. Introduction

The last few decades have witnessed efforts towards eliminating contaminants in food and feed systems, and attaining global food and feed safety, which has necessitated a multidisciplinary approach that involves farmers, scientists, processors, regulators, consumers, governments, and other stakeholders. Food contaminants are categorised into three major groups, including biological, chemical, and physical contaminants. While all contaminants are not to be neglected, biological contaminants pose a serious issue, and are a common cause of food poisoning, spoilage, and loss. Major causes of biological contamination in foods include fungi, bacteria, and viruses. These organisms inhabit our environment and can contaminate food at every stage of production and processing.

The association of fungi with human illnesses have been known for centuries probably even by the Greeks and Romans [1]. Reports from the Middle Ages in European countries revealed an outbreak of ergotoxicosis (St. Anthony’s fire). The outbreak occurred as a result of intoxication with ergoline alkaloids caused by ingestion of sclerotia of *Claviceps purpurea* in rye, following the increase in consumption of rye in the region [2]. In 1960, another major outbreak (Turkey “X”) in birds was recorded in England as a result of the consumption of contaminated feeds leading to the death of 100,000 turkey poults and other domestic fowls such as ducklings and chickens. Turkey “X” disease was later linked to *Aspergillus* fungi (*A. flavus* and *A. parasiticus*) and their secondary metabolites—aflatoxins [3].

Besides *Claviceps* and *Aspergillus* species, other important fungi genera such as *Fusarium*, *Penicillium*, and *Alternaria* with pathogenic and toxigenic potentials have been reported in various agricultural commodities and food products. The frequency of these fungi in a geographical environment is determined by the environmental conditions. While *Fusarium* species are associated with the temperate regions, studies have shown that *Aspergillus* species thrive optimally in tropical regions. Unfortunately, this prediction seems to fluctuate with global climate change [4,5]. Fungi can be pathogenic to plants causing various diseases, while having the capacity (mycotoxigenic) to produce a wide range of small organic toxic compounds known as mycotoxins. Although there are hundreds of mycotoxins existing, the most frequently occurring in agricultural, food and feed products include the aflatoxins, fumonisins, trichothecenes, ochratoxins, zearalenone and their metabolites [6,7,8,9,10,11,12,13,14]. Mycotoxins exhibit various toxic health effects on humans and animals. The health risks range from acute effects, which are characterised by swift and obvious toxic manifestations including death, to chronic effects due to long-term exposure of individuals to mycotoxins. The severity of the toxic effect of a particular mycotoxin depends on the extent of exposure but may also be influenced by some determinant factors which include co-exposure to multiple mycotoxins, age, as well as the health condition of the individual.

Considering that mycotoxins are naturally occurring substances and extremely difficult to completely eliminate in food and feed systems, more proactive measures toward combating and controlling the risk of contamination becomes vital. Most countries and regions have therefore established safety regulations to limit exposure and reduce both direct and indirect risk to the populace. Recently, in a bid to improve the status-quo, most regions such as the European Union (EU) unified their mycotoxins regulatory standards, superseding the various country regulations [15] and improving effective enforcement and regulations by regulatory bodies. However, Africa and other developing continents such as Asia face enormous challenge in this respect. The fragmentation of regulations in Africa especially, has contributed to huge economic losses such as reduced revenue/loss of trade, as well as negative impacts on human and animal health.

Mycotoxin regulations are established based on knowledge of the distribution of mycotoxin concentrations within commodities, availability of exposure and toxigenic data [15]. Generating these data lies in the availability of research funding, technological and analytical facilities, as well as a trained work force, which are often inadequate or not available in African countries. The socio-economic situation of the region including political considerations, food insecurity, poverty, limited/lack of awareness, and trade interests further complicate the situation. The region therefore depends greatly on the regulatory standards set by other regions, irrespective of the wide disparity in cultural, social, and feeding habits that exist among the regions. Until this date, only aflatoxins are regulated in most African countries [16], despite the existence of other mycotoxins in the region. Even in most cases, the existing aflatoxin regulations are only considered for commodities with trade values leaving the local population with unsafe agricultural products. Hence, there are still outbreaks of mycotoxicosis in African countries even in the 21st century.

Whereas there is reported evidence of episodes of acute aflatoxicosis, especially in East Africa between 2001 and 2019, the most severe was reported in 2004 in Eastern and Central Provinces of Kenya. The outbreak was linked to the consumption of aflatoxin contaminated maize, resulting in the poisoning of over 300 people and 125 deaths [17,18,19,20,21]. The most recent aflatoxicosis outbreak occurred in 2016 in the Dodoma and Manyara regions of Tanzania. According to an epidemiological survey, consumption of aflatoxin contaminated maize was implicated as the cause of the outbreak, resulting in the death of about 20 persons out of the 68 people implicated [22]. Consequently, it is necessary and timely to re-examine the cause, status and control of fungi and mycotoxin contamination in Africa. The last decades have witnessed fragmented efforts towards combating contaminants in food and feed systems. There is an urgent need to apply a holistic approach in tackling fungi and mycotoxin related food safety problems amid the imminent social, political, and environmental challenges in the region. One of the strategic approaches towards addressing this problem is the setting, implementation, and enforcement of comprehensive food safety/mycotoxin regulations towards improving public health in the region. The present review aims to examine the status of mycotoxin regulations in Africa with emphasis on the effect of weak/lack of mycotoxin regulatory enforcement on the agriculture, trade, and health sectors. Furthermore, the challenges and limitations hindering the progress and the control of mycotoxins in the region are herein reported.

## 2. Food Safety Management in Africa

Food safety aims to protect consumers from exposure to food contaminants including biological, chemical, and physical hazards through conscious acts of handling food from the field to fork, thereby preventing food borne illnesses, disease outbreaks and death. Safe food promotes health and saves lives, while ensuring food security and attainment of the United Nations sustainable development goals (SDGs), especially goals one to three (no poverty, zero hunger, and good health and well-being). This course lies on the shoulders of both the private and public sectors including individual consumers, traders, food producers and processors, as well as governments and regulatory agencies at both national and international levels. Food safety is vital in the modernization of national food systems and acts as a major determinant in a country’s favourable integration into the global market [23]. In the light of the interwoven relationship between food safety and food security, the Rome Declaration on World Food Security highlights food safety as a major contributory player to obtaining a food secured world [24].

Although efforts toward achieving global food safety are on the increase, changes in environmental conditions, consumer preferences and habits, and new and emerging microorganisms and toxins still pose serious challenges to food safety in the 21st century. A recent World Bank study reported that an estimate of about 110 billion US dollars is lost annually in low- and middle-income countries as a result of food safety problems attributed to lost in productivity and medical expenses of which Africa contributes a reasonable proportion, amounting to tens of billions of US dollars [23]. To date, Africa continues to experience the highest per-capita rate of foodborne illnesses globally due to exposure to food safety hazards. The World Health Organisation (WHO) reported that about 91 million acute illnesses and 137,000 deaths occur in Africa annually, due to foodborne hazards, with the most vulnerable being children, pregnant women, and the elderly [25].

While Africa is the most affected by food safety issues, it is regrettable that this region has the least comprehensive food safety management programmes and lacks prioritization of investments. The problem is worsened by climate change, nonchalant attitudes among governmental organisations and stakeholders, and the complexity of African food supply chains mainly characterised by subsistence farmers, small-scale operators, and unorganised market systems (street markets). The intricacy of food safety in the African continent is often undermined by ignorance and negligence among the populace including food producers, handlers, and consumers, as well as nutritional challenges as a result of food insecurity ravaging the region. Other “most important” pressing issues such as combating malaria, communicable diseases, political instability, insecurity, and natural disasters in most African countries further exacerbates the situation. Unfortunately, existing efforts to achieve food safety are often channelled toward ensuring safe foods for export. There is need for African governments to take a holistic evaluation of food safety standards instead of concentrating mainly on export markets and neglecting the local markets, domestic industries, and local food systems.

Among food safety issues, mycotoxin contamination is ranked as one of the major hazards preventing the African continent from achieving food security. Although efforts such as use of fungi resistant varieties, biological and chemical control agents, improved drying and good storage conditions, have been used by farmers in the region, fungi infection and mycotoxin contamination still cause huge economic losses. According to FAO, an annual estimate of about 4.5 billion people are exposed to mycotoxin contamination, especially aflatoxins, through ingestion [26]. Although the incidence of mycotoxicosis outbreaks such as ergotism and aflatoxicosis have been reported in different parts of the world including Europe and Asia with a low mortality rate, in recent years, mycotoxicosis outbreaks in Africa often result in a high mortality rate in the affected population. An earlier study attributed a case of acute hepatic disease in a Ugandan boy to aflatoxin intoxication caused by ingestion of aflatoxin B1 (1700 µg/kg) contaminated cassava [27]. In 1978, an outbreak of ergotism occurred in Waro and Gazo-Belay sub-Woredas, Wedla-Delanta and Lasta Awrajas, Wollo administrative region of Ethiopia due to the consumption of grains contaminated with fungi (*Claviceps purpurea*), which resulted in about 47 deaths [28,29]. Ngindu et al. also reported an outbreak of aflatoxicosis in the Machakos district of Kenya in 1981 owing to consumption of aflatoxin contaminated maize with a mortality of over 60% [30].

Even in the 21st century, the problem of fungal metabolites in African crops and food products persist. The death of 12 people in Meru North district of Kenya in 2001 was attributed to the consumption of aflatoxin contaminated maize [31]. Three years after, a more severe aflatoxicosis outbreak occurred in Eastern and Central Provinces of Kenya, which resulted in 39% mortality rate out of 317 affected cases [21]. Unfortunately, the outbreak of aflatoxicoses continued in subsequent years (2005–2008 and 2010) especially among the rural subsistence East African farmers [32]. A comprehensive study in Eastern Kenya reported that about 477 poisoning occurred between 2004 to 2010 due to intake of aflatoxin contaminated foods resulting in 40% fatality rate [33]. Recently an outbreak of aflatoxicosis was reported between May and November 2016 in Manyara and Dodoma regions of Central Tanzania with symptoms of jaundice, vomiting, abdominal distension, diarrhoea, swelling of lower limbs, headache, and fever, leading to 30% fatality [22]. Another outbreak episode of aflatoxicosis linked to the consumption of mouldy maize that killed four children in the Kiteto District of Tanzania between June and July of 2017 was also reported [34].

While some of these cases were documented, the possibility of these figures being underestimated is high due to inadequate and unorganised coordinated monitoring system and medical surveillance, which often lead to many unreported cases. It is pertinent to note that only outbreaks of mycotoxicosis due to aflatoxins have been documented in Africa, notwithstanding the wide range of other mycotoxins contaminating food stuff in the region. Surprisingly, these outbreaks originated from only one region—Eastern Africa. Could this be as a result of the peculiarity of the environmental conditions, institutional challenges, food insecurity, and mycotoxin concentration in the staple foods, or food preferences and habits in the region when compared to other regions of Africa? The fact that other regions of Africa share the same environmental and institutional conditions suggests that East Africa is probably more proactive than other regions as regards to food safety alert systems for mycotoxins.

In addition, human exposure to aflatoxins have been associated with a high incidence of hepatocellular carcinoma (HCC) in Africa, especially in sub-Saharan Africa (SSA), with individuals infected with hepatitis B virus (HBV) being at higher risk [35]. Zain attributed about 250,000 annual deaths caused by HCC in China and SSA to be linked to aflatoxin exposure [36]. A practical example is reflected in a Tanzanian study in 2014, which estimated that about 3334 cases of HCC in Tanzania were caused by aflatoxin contamination and resulted in 95% death, and 96,686 DALY (disability adjusted life years) [37]. This report is in line with the findings of a country-led situation analysis conducted by the Partnership for Aflatoxin Control in Africa (PACA) in Tanzania, which estimated that about 4825 new cases of aflatoxin-induced liver cancer occur annually, based on the estimated aflatoxin exposure rate (10,926 ng/kg body weight (bw)/day) and HBV prevalence (7.07%) in the population [38]. The same trend was reported for Gambia, Malawi, Nigeria, Uganda and Senegal with estimated annual new aflatoxin-induced liver cancer cases of 160, 2171, 3262, 3700, and 4118, respectively, based on each country’s estimated aflatoxin exposure rate (155, 261, 34.8, 266, and 416 ng/kg bw/day) and HBV prevalence (15, 12, 14, 10, and 12%), respectively [39,40,41,42,43].

Among other mycotoxins, fumonisin B1, the most prevalent of the fumonisins have been postulated to have a potential role in the aetiology of human oesophageal cancer (OC) [44]. Although this toxin is classified as a possible carcinogen (group 2B carcinogen) to humans by the International Agency for Research on Cancer (IARC) because of the uncertainty in the mechanisms of its carcinogenesis [45], Marasas further associated the high incidence of OC in the Transkei region of South Africa to an occurrence of high levels of fumonisin B1 in home grown-staple maize consumed on a daily basis in the region [44]. A recent study also alleged human exposure to fumonisins is a major contributing factor to a high prevalence of OC among the Kalenjin community in Western Kenya [46]. Evidence of developmental and other health problems including child stunting, immune dysfunction, renal toxicity, estrogenic effects, inhibition of protein and ribonucleic acid synthesis associated with mycotoxins including zearalenone, ochratoxin A, and deoxynivalenol have also been reported in Africa and in other parts of the world [47,48,49,50,51,52].

As public awareness of the consequences of mycotoxin contamination of food in the African continent is increasing, food safety approaches in the region need to be strategic, comprehensive, and well-coordinated. During the seventh partnership platform organised by the Comprehensive Africa Agriculture Development Programme (CAADP) in 2011, participants decided to establish an African Working Group under the directive of the African Union Commission (AUC) for aflatoxin control. Based on this advice, an innovative consortium—Partnership for Aflatoxin Control in Africa (PACA) was established in 2012 with the vision to create “an Africa free from the harmful effects of aflatoxins” through coordinated approaches which are aimed at mitigating, controlling, and managing aflatoxins across the agriculture, health, and trade sectors, thus safeguarding consumers’ health, and facilitating trade. PACA in collaboration with the Economic Community for West African States (ECOWAS) and other stakeholders across Africa in 2014 established a comprehensive Africa-wide approach to mitigate the impact of aflatoxins on agriculture, food security, trade, and health [53]. With ECOWAS at the forefront, PACA and other stakeholders including the International Institute of Tropical Agriculture (IITA), Forum for Agricultural Research in Africa (FARA) and other partners, developed a regional action plan to identify vital actionable strategic interventions that are applicable across ECOWAS member states.

In the efforts of PACA, a unique management tool known as Africa Aflatoxin Information Management System (AfricaAIMS) was established in six pilot African countries including Gambia, Malawi, Nigeria, Senegal, Tanzania, and Uganda with the duty to generate and provide comprehensive data on aflatoxin contamination of agricultural food products and animal feeds, as well as on other aflatoxin related issues in the health and trade sectors. The initiative’s key objective is to provide locally relevant, home grown and reliable evidence to facilitate informed decisions on policies, food safety regulations and standards, mitigation interventions such as educational and technological, resource allocation, and advocacy and awareness raising activities by the government and other stakeholders [41,54]. PACA also provided catalytic support to develop a resource mobilization strategy and convene business meetings to enhance ownership and financing of the national aflatoxin control plan. This support also extends to convening aflatoxin working groups to spearhead planning and implementation of aflatoxin mitigation actions at the country level [41].

In a bid to improve trade among countries in Eastern Africa, a Bureau of Standards was formed with a sole mandate to harmonise standards for goods and services and set regulatory limits for mycotoxins in foods and feedstuff in the region [55]. Other regional platforms such as Permanent Interstate Committee for drought control in the Sahel (Comité permanent inter-État de lutte contre la sécheresse au Sahel (CILSS)) and Common Market for Eastern and Southern Africa (COMESA), as well as regional governments and governmental institutions have also geared efforts toward creating awareness, management and control of aflatoxins in their respective regions [56]. Although there is increasing momentum in Africa towards aflatoxin management and control, these efforts, as reported earlier, are challenged by poor infrastructure, lack of political commitment, complex food chain systems, and stringent market mechanisms. Approaches including policies, legislation, and conscious implementation of food safety management systems needs to be prioritized, which will encompass other forms of mycotoxins that frequently occur in agricultural and food products in the region with the aim of protecting over 1.216 billion people in the region.

## 3. Status of Mycotoxin Regulation in Africa

As elimination of mycotoxins from agricultural, food, and feed commodities is virtually impossible, national and/or international regulatory limits are set, incorporated in food control systems, and serve as a benchmark to monitor and regulate these toxic substances in food and feed products. To effectively protect consumers from exposure to food hazards, food control system must be efficacious. The establishment and implementation of regulatory limits for mycotoxins in Africa is faced with several hurdles because of food insecurity. Another factor is the trajectory of the farming system in the continent. Africa’s major agricultural production is controlled by the rural subsistence farmers, who grow agricultural produce mainly for self-consumption, and have little or no awareness on mycotoxin contamination. While there is evidence of improvement in the establishment of the mycotoxin limits, especially for aflatoxins between 1990 and early 2000 in Africa, there is still need for more concerted efforts.

According to FAO, only 28% (15 countries) out of 54 African countries were known to have mycotoxin regulations by 2003 [16]. It is worrisome that, except for Morocco that has updated its mycotoxins regulation [57], other countries have maintained the status quo. Regrettably, even in some of the countries with mycotoxin regulations, it is often restricted to international markets. For instance, in Malawi, the only existing mycotoxin regulatory limit is for peanuts meant for export, regardless of the frequent occurrence of these toxins in peanuts [58], which serves as a major source of daily nutrients to large numbers of the population. This fact was also revealed by a study in Côte d’Ivoire, which highlighted that research on mycotoxins is mainly focused on cocoa and coffee because of their economic importance in the country as cash crops [59]. The consequence of this exclusive attempt in the control of mycotoxins on the economy and health of the local populace cannot be understated. This act implies that the best of the agricultural produce is preserved by farmers for the international markets for economic gains, while exposing the local population to poor quality produce. However, it could also lead to inducement and improvement of strategic steps throughout the agricultural and food systems for reduction in fungi infestation and mycotoxin contamination in food products.

As mentioned earlier, Morocco has the most comprehensive and updated mycotoxin regulation (Table 1), comparable to that of the EU in the African continent, which encompasses aflatoxins, fumonisins, ochratoxin A, deoxynivalenone, patulin and zearalenone in a wide range of primary agricultural products and foodstuffs [57]. Unfortunately, South Africa, a country that pioneered research on fumonisins, with proven reports on the frequent occurrence and high concentration of these toxins in agricultural and food products to date has no established regulatory limit for fumonisins [60]. Similarly, the Standard Organisation of Nigeria (SON) has set regulatory limits of 10, 20, 4, 4 and 10 µg/kg for total aflatoxins in maize grains, raw groundnuts, kulikuli (groundnut cake), sesame seeds and sorghum respectively [41,61]. Nevertheless, some countries have adopted the EU mycotoxin regulatory limits toward responding to the needs of international trade. As of 2003, Nigeria set regulatory limit of AFB1 at 20 µg/kg in foodstuffs but have in recent times adopted the EU limits for aflatoxin B1 (2 µg/kg) and total aflatoxins (4 µg/kg) for maize with the intention to optimize trade gain.

**Figure 1:**
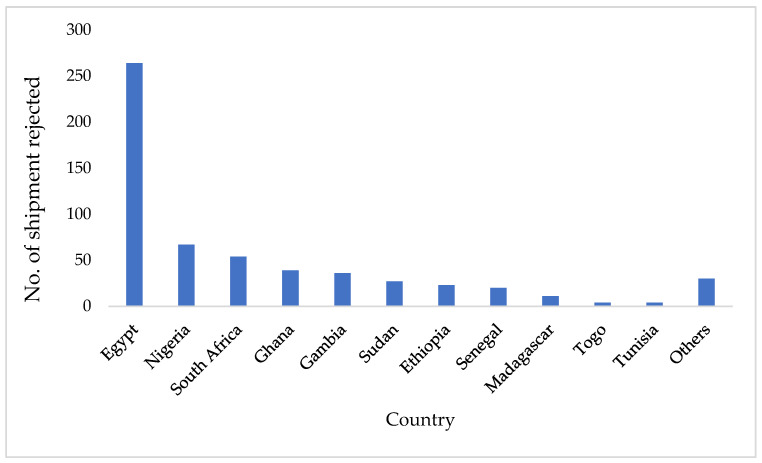
EU rejection of African products (groundnut and groundnut products, bitter almond, mixed spices, chilli powder, melon seed, suya pepper) due to mycotoxin contamination from 2005 to 2020. Source: RASFF Online Database. Others include countries (DR Congo, Congo, Angola, Kenya, Malawi, Mauritius, Tanzania, and Uganda) that each had one EU rejection between 2005 and 2020.

Notwithstanding, the conscious practice to control mycotoxin contamination and eliminate its impact on the economy targeting international trade, the financial burden caused by the rejection of African food products due to non-compliance with mycotoxins standards still persist. According to the European Commission (EC) Rapid Alert System (RASFF), mycotoxins contribute an average of 39% of total EU annual border rejection experiences in African food and feed systems. The implication of these rejections results in loss of the country’s reputation and reduction in the earnings from exports through loss of exported products, transportation costs, and logistics and insurance costs. Between 2005 and 2020, 579 shipments of agricultural products of African origin were rejected by the European Union due to mycotoxin contamination, especially aflatoxins above the EU legislative limits (groundnut and all cereal: 4 µg/kg total aflatoxins), with Egypt (264), Nigeria (67) and South Africa (54) contributing the highest number (Figure 1 and Table 2). The numbers of border rejections are a result of non-compliance to EU standards and/or non-standardised analytical protocols among the African countries and the European Union. Groundnut and groundnut-based products were the most affected products (88%). It is also noteworthy that border rejection within the African continent is a common incident. For example, despite the harmonization of regulatory limits for mycotoxins in food and feedstuff in the East African Community (EAC), there have been trade conflicts among countries in the region owing to extremely high concentrations of aflatoxins in staple crops. The most recent is the Kenyan ban on maize imports from Uganda and Tanzania as a result of aflatoxin contamination above the East Africa Community safety limits, thus resulting in economic losses [66].

While some countries in the region do not have national mycotoxin regulatory limits, it is important to mention that the majority of these countries are members of the Codex Alimentarius Commission (CAC), who through the establishment of international standards, guidelines and codes of conducts aim to facilitate world trade, thus protecting consumers. All the Codex member countries can adopt and operate within the gambit of the Codex standards. In addition, the African Organisation for Standardization, which is an African intergovernmental organisation made up of the Organisation of African Unity (AU) and the United Nations Economic Commission for Africa (UNECA) have set mycotoxin regulatory limits (total aflatoxins—10 µg/kg, aflatoxin B1—5 µg/kg, fumonisin—2000 µg/kg) for maize grains because of the importance of maize crops in the region [67]. Based on Codex, AU, and UNECA, the 54 African countries therefore have some form of mycotoxin regulatory limits either by establishment of national standards or by proxy.

## 4. Challenges in Setting and Enforcing Mycotoxin Legislation

In most countries, issues that involve food safety regulations are controlled and managed by stakeholders, which often include scientists, farmers, marketers, processors, consumers, policy makers and the government, while taking the international standards and several other factors such as food security and food culture into consideration. Due to the benefits associated with the control and monitoring of mycotoxins in agricultural and food products, there has been an increase in awareness of fungi and mycotoxin contamination, as well as improved efforts by regulatory bodies towards setting limits to protect consumers. Despite efforts geared toward achieving mycotoxin free agricultural commodities, factors such as insufficient and/or unavailability of scientific data, food insecurity, complex food supply chains, and lack of awareness, still limit the absolute control of mycotoxins and their producing organisms, especially in Africa.

The establishment of mycotoxin regulatory limits and enforcement seems to have undergone significant transformation since the 2003 worldwide mycotoxin regulations [16] as most of the African governments have intensified efforts to improve food safety by reviewing and updating key components of the national food safety control system [68]. However, these efforts still face numerous challenges, hindering the establishment of mycotoxin regulatory limits and enforcement of the existing ones, and even making the adoption of Codex laws and African standards for countries that have no mycotoxin regulatory limits ineffective.

### 4.1. Scientific Limitations

Scientific research remains one of the prerequisites for human and societal development as it directly or indirectly influences the standard of life worldwide. Science plays a major role in providing the foundational information to which mycotoxin limits are set, including procedures for sampling and analytical methodology, mycotoxin occurrence data and maps of various agricultural commodities and food products, exposure data and toxicological data. The availability of reliable data is dependent on the validity of the sampling protocol and analytical methods. The distribution of mycotoxins in commodities is often heterogeneous in nature because of biotic and abiotic variables, hence precautionary efforts should be put in place during sampling in order to acquire representative samples. Furthermore, accessibility of research infrastructures, research funding and technical manpower is very crucial for the establishment of regulatory laws. While in recent times there has been an increase in training and capacity building for African scientists and collaboration with scientists in developing countries, these trainings and opportunities have little impact on scientists in the region. One of the major obstacles to this is the brain drain. Although there are no reliable data on brain drain in Africa, the region records the highest rate of brain drain when compared to other parts of the world. In 2020, the index points were between 4.3 (Mauritius) and 8.9 (Somalia) [69]. This is the resultant effect of a dearth of satisfactory work conditions, lack of funding, lack of research infrastructures and limited allocation of resources for science in Africa.

In most of the African countries, it is practically impossible to carry out a whole innovative research study due to the lack of a standard laboratory. This is evident in mycotoxin research, highlighting the reason why the majority of the studies on mycotoxins emanating from the region are mostly done as a collaborative study using the facilities of laboratories in developed regions especially Europe and North America [70,71,72,73,74,75,76]. Even when such studies are done solely in Africa, the reliability and reproducibility of the data produced is often questioned because of the analytical methods used. Recent advancements in instrumentation, such as liquid chromatography-mass spectrometry (LC-MS) and high-resolution mass spectrometry (HR-MS), research methodology, means research facilities require huge funding. It becomes imperative for government and stakeholder to prioritise research while establishing technical outlets for manufacturing companies and technical offices in the region for easy procurement of instruments, and availability and accessibility of professional servicing personnel within the region.

In addition, developing technical expertise of African origin with respect to maintenance and management of these sensitive analytical instruments will be more pro-active, and in the long run more beneficial, leading to increases in research output [68]. Moreover, training of scientists with the capacity to develop cheap, rapid, sensitive, precise, and selective test methods may be an alternative to solving the problem of instrumentation. African scientists are often cut out of current global research trends because of technology gaps resulting from a lack or limited availability of high-speed internet, which is vital for accessing scientific books, journals, and databases [77]. Other challenges such as epileptic power supplies in most African countries, makes it virtually impossible to carry out mycotoxin research in the region because most of the materials required for the studies need to be stored at low temperatures, thus limiting the scope and reliability of studies done.

### 4.2. Food Insecurity

Food security implies that all people at all times, have physical, social, and economic access to sufficient, safe, and nutritious food that meets their food preferences and dietary needs for an active and healthy life [78]. Food safety cannot be disassociated from food security, as exposure to unsafe foods remains the major hurdle hindering the attainment of a food secured world and United Nation Sustainable Development Goals (SDGs), especially in developing countries. Several factors play significant contributions to food insecurity; however, extreme climate change and economic downturn remain the foremost drivers in Africa. The impact of conflict resulting in displacement of people, inability of farmers to access farms/fields and markets in several parts of the region has complicated the scenario. In addition, the Technical Centre for Agricultural and Rural Cooperation clearly emphasises the detrimental effect of mycotoxins in achieving food security and food safety in Africa [79]. The occurrence of mycotoxins in agricultural commodities and food products in Africa has been associated with high rates of post-harvest losses in the region, which is detrimental to the economic and health sectors and can lead to death in humans and animals [36,80]. According to 2018 data, about 277 million people are hungry in Africa with about 239 million of this population inhabiting sub-Saharan Africa [81]. In addition to the population exposed to severe food insecurity, about 676.1 million of the total population of Africa are within moderate to severe food insecurity, which implies that Africa contributes 33.57% to the 2013.8 million world moderate or severe food insecure population [81].

Given the obvious evidence of food shortage, hunger, and malnutrition in the region, it is difficult to set and enforce stringent mycotoxin regulatory limits when a reasonable proportion of the African population lavish in hunger. What is the possible way out of this problem? While low quality food products are often contaminated and unsafe for human consumption, the socio-economically deprived African population leverage on these products for survival because of their affordability. This suggests that a large number of the Africans living in extreme poverty may be exposed to these toxins. Therefore, setting strict mycotoxin regulatory limits for economically challenged African populations will result in the loss of a large proportion of food, therefore leading to an increase in food shortages in the population. In a survey of Nigerian cereals in 2015 and 2016, marketers reported that bad quality cereals (fungi contaminated) are often fed to chickens or reserved for food processors for further processing in order to maximise profits [71,82]. Although processing has been reported to reduce mycotoxin concentration in food products [82,83,84], the problem of possible modification of these toxins to other forms that are often not analysed and quantified in food products, should not be ignored. In our opinion, addressing the problem of mycotoxins should be multifaceted. While setting regulatory standards for these toxins is very important, the socio-economic status of the African population is of utmost importance and influences the enforcement of the set laws.

### 4.3. Social-Cultural Limitations

“Social and cultural standards are rules or expectations of behaviour within a specific cultural or social group” [85]. These standards may be a positive or negative influence on the behaviour of an individual, thus governing and regulating one’s beliefs and interactions with others. The African continent has embedded styles and traditions of life. Socio-cultural norms play a crucial role in the selectivity and acceptability of foods by the people and have the potential to influence the food systems, thus, should be considered when setting food safety laws and regulations including mycotoxin legislations.

Africa has the most complex food supply chain system, affected by poor structure and technology, government, and societal structure. Beyond these factors, food supply chain management players should not be alienated from the barriers that affects food supply chains in Africa. Contamination of agricultural and food products by fungi and mycotoxins may occur at any stage in the food supply chain, thus identifying the appropriate sampling point of agricultural and food products becomes difficult. Therefore, organisation and cooperation among stakeholders within the food safety chain becomes a tool towards achieving reliable sampling and scientific data generation.

Furthermore, studies have shown that some agricultural crops are more susceptible to fungi infections and mycotoxin contamination such as the cereal crops [71,86]. Of the cereal and legume crops, maize and groundnut, and their products have been reported as being more susceptible to mycotoxin contamination including *Aspergillus* and *Fusarium* produced mycotoxins such as aflatoxins and fumonisins [73,75,87,88]. Culturally, East and Southern Africa grow a wide variety of cereal crops, but the main staple crop in the region is maize, which is eaten on a daily basis, often with groundnut butter, thus increasing the risk of exposure to mycotoxins. Consequently, mycotoxin regulatory limits in maize should be at the lowest value. Ironically, only a few countries in the region have limits for aflatoxin contamination in maize, which are higher compared to the European limit for aflatoxins (Table 1). Besides, there are no regulatory limits for other mycotoxins (fumonisins, deoxynivalenol, zearalenone) often contaminating maize cereal in the region.

In view of this, dietary diversification (substituting maize with other cereals) could be an option towards reducing mycotoxin exposure in the population. Wu and co-workers highlighted that an increase in dietary diversity can play a potential role in reducing consumption of toxins and increase the intake of nutrients that could counteract the toxicity of toxins, thus preventing disease [89]. This report is supported by a Chinese study, which reported that substituting maize consumption with rice and other food stuffs, remarkably reduced exposure to aflatoxins (causative agent of liver cancer), and subsequently decreased the rate of mortality caused by liver cancer in Qidong, China [90]. While changes in food consumption patterns and practices seems challenging, this strategic intervention was actualized in Qidong China because of the reforms in the Chinese food policies facilitated by the government, thus suggesting that the government has a great role to play in the modification of social-cultural behaviour of the population.

## 5. Conclusions and Future Perspectives

Production of healthy foods and sustenance of livelihoods should be a top priority in Africa. Safe food production is feasible within a multidisciplinary context involving food producers, processors, food scientists, technologists, toxicologists, food regulators and policy experts. Food safety tends to be accorded very little policy attention and investment, and only comes into the limelight in the course of foodborne disease crises. The agricultural food system and market is presently stretched with a dramatic increase in human population and lifestyle changes including urbanization and income growth which results in diet changes with more dispositions to processed foods. Consequently, it is projected that intra African food demand will rise by 178% by 2050 [91]. Improving the status of food security while ensuring environmental sustainability through interventions that better human health, need to be prioritized.

Agriculture, nutrition, and health overlap and provide opportunities for policy makers and development experts. These opportunities are embedded in harnessing capacities in terms of adaptation, evaluation, and use of innovative tools in linking the three aforementioned sectors within the framework of research policies and practice. In principle, optimizing the potentials of bio-fortification while promoting safe agricultural and food systems practices that reduce infectious and chronic disease risk is encouraged. Africa has the highest per capita incidences of foodborne diseases and deaths, with fungi and mycotoxins, especially aflatoxins, being one of the leading causes of foodborne deaths [25]. These diseases create huge economic losses of around 110 billion US dollars in terms of production loss and medical costs. Notwithstanding the huge burden of health costs and the loss of productivity associated with food-borne diseases, alterations and shifts in food marketing brings up yet another negative dimension.

A concise view of the status quo suggests the recurring problems of inadequate laboratory testing equipment and limited capacity to identify food safety benchmarks. This is significantly linked to low investment, non-establishment of national food standards, and negligence of existing international standards. The bureaucratic, poor incentive working conditions lead to weakness in the monitoring systems by enforcement and regulatory agencies. In addition, the African traditional food systems result in unregulated trade deals within and beyond communities. The African Continental Free Trade Agreement (AfCFTA) seeks to boost intraregional trade of agrifood products within 54 countries. This will usher in a large trade platform and presents a huge opportunity for the growth of Africa. This agreement suggests that countries must work to harmonize their sanitary and phytosanitary regulations in line with international science-based standards. The African Union (AU) recently adopted a sanitary and phytosanitary (SPS) policy framework in line with World Trade Organisation (WTO) obligations that will help Member States in harmonizing and strengthening of their SPS measures within the AfCFTA. It is noteworthy to highlight the Malabo declaration by leaders of African nations. It was agreed that countries must document gains in food safety and production with a focal aim of linking food safety with agricultural development.

Based on the aforementioned context, challenges and huge opportunities, it is imperative to sustain the political and power framework that will ensure sustainable legislative and governance backing within African food systems. This can be done through engagement of politicians and decision makers. Beyond this, integrated data systems and platforms need to evolve for improved efficiency in risk and hazard assessment. This evidence-based decision making will lead to optimal use of resources while enhancing the operational performance and capacity delivery of food control systems. Active networking is needed by African food safety stakeholders with more presence at international meetings. Improving linkages through enhanced channels for information dissemination in food systems and cultures should be prioritized within African countries. This will have a resultant effect in raising awareness that will thus stimulate solutions to existing problems. End users and consumers need to be empowered with the right knowledge with regards to healthy and safe food choices. This will translate to improved consumer/end user and industry awareness of food safety, thereby generating demand for reforms at institutional levels of legislation, governance, and infrastructure investment. The standardization of protocols by regulatory agencies across countries should be accorded utmost priority. This can be improved with more synergistic coordination, thus improving border regulatory systems that facilitates safe trade of food products. From a regulatory perspective, safe food implies food products with an appropriate level of protection [92].

The aims and purposes of food legislation is to protect and sustain livelihoods of the end users, protection of the consumers from being defrauded, while ensuring standardized quality and wholesomeness of foods products. Existing legislative frameworks in most African countries are mostly rudimentary, archaic without sound scientific backing [93]. For legislations to benefit intended beneficiaries the standards are meant to be science-based as required by Codex. It becomes imperative that a sustainable and working legislative framework should encompass a pre-emptive approach with significant focus on facilitating the delivery, processing, and distribution of safe food, while placing less emphasis on penalties. In addition, efforts to align food commerce with the demands of WTO and the Codex standards and codes of practice are highly encouraged. Different models of regulatory enforcements have been put in place in many developed countries. An essential component that cuts across the flexibilities of enforcement models must include the following within the stakeholders: transparency, inclusiveness, integrity, clarity of roles, accountability, science/risk-based approach [93]. Effective food safety management and legislation should therefore improve investment, lead to better regulatory capability, and a firm understanding of behavioural dynamics and shared responsibilities within government, food business managers, and consumers.

## Figures and Tables

**Table 1:** Mycotoxins regulatory limits in foodstuff in Africa.

Country	Mycotoxin Type	Commodity	Limits (µg/kg)	Reference
Algeria	AFB1	Peanuts, nuts, cereals	10	[16]
	AFB1, B2, G1, G2	Peanuts, nuts, cereals	20	
Botswana	Aflatoxins	All foods	15	[16]
EAC	AFB1	Selected foods, cereals, pulses	5	[62,63]
	AFB1, B2, G1, G2	Selected foods, cereals, pulses	10	
	AFM1	Milk	0.05	
Egypt	AFB1	Peanuts and cereals	5	[16]
	AFB1, B2, G1, G2	Peanuts and cereals	10	
	AFB1	Maize	10	
	AFB1, B2, G1, G2	Maize	20	
	AFM1	Milk intended for adults	0.5	
	OTA	Coffee	5	
	DON	Wheat and wheat flour	700	
		Barley and barley flour	1000	
Malawi	AFB1, B2, G1, G2	Peanuts	3	[40,64,65]
Mauritius	AFB1	Peanuts, other foods	5	[16]
	Aflatoxins	Peanuts/other foods	15/10	
Morocco	AFB1	(1.1). Peanuts and other oilseeds, hazelnuts and walnuts intended for sorting or other physical methods before human consumption or use as an ingredient in food products, unless they are intended to be crushed for the manufacture of refined vegetable oil	8	[57]
	AFB1, B2, G1, G2		15	
	AFB1	(1.2). Almonds, pistachios and apricot kernels intended for sorting or other physical methods before human consumption or use as food ingredients	12	
	AFB1, B2, G1, G2		15	
	AFB1	(1.3). Other nuts (except nuts listed in 1 and 2) intended for sorting or other physical methods before human consumption or use as food ingredient	5	
	AFB1, B2, G1, G2		10	
	AFB1	(1.4). Peanuts and other oilseeds and their products intended for direct human consumption or use as ingredients for food products, with the exception of crude vegetable oils intended to be refined and refined vegetable oils	2	
	AFB1, B2, G1, G2		4	
	AFB1	(1.5). Almonds, pistachios and apricot kernels intended for direct human consumption or use as food ingredients	8	
	AFB1, B2, G1, G2		10	
	AFB1	(1.6). Hazelnuts and Brazil nuts for direct human consumption or use as an ingredient in foodstuffs	5	
	AFB1, B2, G1, G2		10	
	AFB1	(1.7). Nuts (except nuts listed in 5 and 6) and their products intended for direct human consumption or for direct use as an ingredient in foodstuffs	2	
	AFB1, B2, G1, G2		4	
	AFB1	(1.8). Dried fruits, other than dried figs intended for sorting or other physical methods before human consumption or use as food ingredients	5	
	AFB1, B2, G1, G2		10	
	AFB1	(1.9). Dried fruits and their products (other than dried figs) intended for direct human consumption or use as food ingredients	2	
	AFB1, B2, G1, G2		4	
	AFB1	(1.10). Dried fruits	6	
	AFB1, B2, G1, G2		10	
	AFB1	(1.11). All cereals and their products, with the exception of food products listed in 1.12, 1.15 and 1.17	2	
	AFB1, B2, G1, G2		4	
	AFB1	(1.12). Maize and rice intended for sorting or other physical methods before human consumption or use as an ingredient for food products	5	
	AFB1, B2, G1, G2		10	
	AFM1	(1.13). Raw milk, heat-treated milk and milk-based products	0.05 *	
	AFB1	(1.14). Spices including *Capsicum* spp. (chili peppers, chilli powder, cayenne pepper and paprika), *Piper* spp. (white pepper and black pepper), *Myristica fragrans* (nutmeg), *Zingiber officinale* (ginger), *Curcuma longa* (Indian saffron), mixtures of spices containing one or more of the spices aforementioned.	5	
	AFB1, B2, G1, G2		10	
	AFB1	(1.15). Cereal-based baby food intended for infants and toddlers	0.10	
	AFM1	(1.16). Infant formulas including infant milk	0.025	
	AFB1	(1.17). Special dietary foods for medical purposes, specifically for infants	0.10	
	AFM1		0.025	
	OTA	(2.1). Raw cereals	5	
		(2.2). All products derived from raw cereals, including processed cereal products and cereals intended for direct human consumption, with the exception of food products listed in points 2.9, 2.10 and 2.14	3	
		(2.3). Raisins (currants, sultanas and others raisins)	10 *	
		(2.4) Roasted coffee beans including ground, except soluble coffee	5	
		(2.5). Instant coffee (instant coffee)	10	
		(2.6). Wines (including sparkling wines, excluding liqueur wines and wines with minimum alcoholic content of 15%) and fruit wines	2 *	
		(2.7). Flavoured wines, flavoured wine-based drinks and cocktails flavoured with wine products	2 *	
		(2.8). Grape juice, grape must, reconstituted concentrated grape juice and grape must, intended for direct human consumption	2 *	
		(2.9). Cereal-based baby food intended for infants and young children	0.5	
		(2.10). Special dietary foods for medical purposes, specifically for infants	0.5	
		(2.11). Spices including dried *Piper* spp. (white pepper and black pepper), *Myristica fragrans* (nutmeg), *Zingiber officinale* (ginger), *Curcuma longa* (Indian saffron), *Capsicum* spp. (chili peppers, chilli powder, chili pepper, Cayenne and paprika), mxed spices containing one of the spices aforementioned	15	
		(2.12). Licorice wood (*Glycyrrhiza glabra*, *Glycyrrhiza inflateet,* other species), ingredient for infusion	20 *	
		(2.13). Licorice extract (*Glycyrrhiza glabra, Glycyrrhiza inflateet,* other species), for use in food products, especially beverages and confectionery	80 *	
		(2.14). Wheat gluten not sold directly to the consumer	8	
	PAT	(3.1). Fruit juices, reconstituted fruit juice concentrates and fruit nectars	50 *	
		(3.2). Spirits, cider and other fermented drinks produced from apples or containing apple juice	50 *	
		(3.3). Products made from apple pieces, such as applesauce and apple puree intended for direct consumption with the exception of food products listed in 3.4 and 3.5	25 *	
		(3.4). Apple juice and products made from apple pieces, such as applesauce and mashed potatoes intended for infants and young children, and labelled and sold as such	10	
		(3.5). Foods for babies, other than cereal-based products intended for infants and children	10	
	DON	(4.1). Raw cereals other than durum wheat, oats, rice and maize	1250 *	
		(4.2). Durum wheat and raw oats	1750 *	
		(4.3). Raw maize except raw maize intended for processing by wet grinding	1750	
		(4.4). Cereals intended for direct human consumption, cereal flour, bran and germ as a finished product marketed for direct human consumption, with the exception of food products listed in 4.7, 4.8, 4.9 and rice products	750 *	
		(4.5). Dry pasta	750 *	
		(4.6). Bread including small baked goods, pastries, cookies, cereal and cereal snacks for breakfast	500 *	
		(4.7). Cereal-based baby food intended for infants and young children	200	
		(4.8). Corn milling fractions including particle size is >500 microns	750	
		(4.9). Corn milling fractions including particle size is ≤500 microns.	1250	
	ZEN	(5.1). Raw cereals other than corn and rice	100 *	
		(5.2). Raw maize except raw maize intended for processing by wet grinding	350	
		(5.3). Cereals intended for direct human consumption, cereal flour, bran and germ as a finished product marketed for direct human consumption, with the exception of food products listed in 5.6, 5.7, 5.8 and 5.9 and rice products	75 *	
		(5.4). Refined corn oil	400 *	
		(5.5). Bread including small baked goods, pastries, cookies, cereal and cereal snacks for breakfast, excluding corn snacks and corn-based breakfast cereals	50 *	
		(5.6). Maize intended for direct human consumption, corn snacks and breakfast cereals	100	
		(5.7). Cereal-based baby food intended for infants and young children	20	
		(5.8). Corn milling fractions including particle size is >500 microns	200	
		(5.9). Corn milling fractions including particle size is ≤500 microns	200	
	FB (sum B1 + B2)	(6.1). Raw maize except raw maize intended for processed by wet grinding	4000	
		(6.1). Maize for direct human consumption, food made from corn for human consumption direct, with the exception of feeds listed in points 2.6.3 and 2.6.4	1000	
		(6.1). Corn-based breakfast cereals and snacks corn-based	800	
		(6.1). Corn preparations and baby food intended for infants and young children	200	
		(6.1). Corn milling fractions including particle size is >500 microns	1400	
		(6.1). Corn milling fractions including particle size is ≤500 microns.	2000	
Mozambique	AFB1, B2, G1, G2	Peanut, peanut milk, peanut butter, maize, cereals and feedstuffs	10	[16]
Nigeria	AFB1	Maize	2	[41,61]
	AFB1, B2, G1, G2	Maize	4	
	AFB1, B2, G1, G2	Sorghum, millet grains	10	
	AFB1, B2, G1, G2	Kuli kuli (groundnut cake), sesame seed, fruits and fruit products, baby and infant foods, tea, coffee and cocoa products, malt drink, wheat flour, composite flour, wheat semolina, shea butter and shea nut kernals	4	
	AFB1, B2, G1, G2	Raw groundnut	20	
	AFM1	Baby and infant foods	0.05	
South Africa	AFB1	All foods	5	[16]
	AFB1, B2, G1, G2	All foods	10	
	PAT	All foods	50	
	AFM1	Milk and milk products	0.05	
Sudan	AFB1, B2, G1, G2	Oil seeds	15	[16]
	Ochratoxin A	Wheat	15	
Tanzania	AFB1	Cereals, oil seeds	5	[16,38]
	AFB1, B2, G1, G2	Cereals, oil seeds	10	
Tunisia	AFB1	All products	2	[16]
Uganda	AFB1, B2, G1, G2	All foods	10	[42]
Zimbabwe	AFB1	All foods	5	[16]
	AFB1	Groundnuts, maize, sorghum	5	
	AFG1	Groundnuts, maize, sorghum	4	

* Alert threshold. The section Morocco: numbers in bracket were used for numbering commodities for the purpose of referencing within text. EAC—East African Community, members of EAC—Burundi, Kenya, Rwanda, South Sudan, Tanzania, and Uganda. African countries with no national regulatory limits—Angola, Benin, Burkina Faso, Burundi, Cameroon, Cape Verde, Central AR, Chad, Comoros, Congo, Democratic RC, Djibouti, Equatorial Guinea, Eritrea, Eswatini, Ethiopia, Gabon, Gambia, Ghana, Guinea, Guinea Bissau, Lesotho, Liberia, Libya, Madagascar, Mali, Mauritania, Namibia, Niger, Rwanda, Sao Tome and P., Seychelles, Sierra Leone, Somalia, South Sudan, Swaziland, Togo, Zambia.

**Table 2:** European border rejection of African products due to mycotoxin contamination between 2018–2020 (compiled from EU RASFF).

Country	Product	Toxin	2018			2019			2020		
			^a^No./Year	^b^No./Product/Year	Conc. µg/kg	^a^No./Year	^b^No./Product/Year	Conc. µg/kg	^a^No./Year	^b^No./Product/Year	Conc. µg/kg
Angola	Groundnut kernel	AFB1	-	-	-	-	-	-	1	1	175.0
		AFT			-			-			190.0
Egypt	Groundnut	AFB1	37	15	7.4–200	35	14	6.8–164.4	19	1	6.8–7.4
		AFT			9.2–230			8.3–191.0			8.2–8.4
	Unshelled Groundnut	AFB1		12	4.7–53.6		19	3.8–42,100		2	110.2
		AFT			6.6–93.4			4.4–46,800			82.4–153.2
	Organic groundnut kernel	AFB1		-	-		1	13.0		14	5.1–11,000
		AFT			-			15.0			12.0–14,000
	Roasted groundnut	AFB1		-	-		1	8.0		-	-
		AFT			-			9.3			-
	Roasted groundnut kernel	AFB1		1	11.6		-	-		-	-
		AFT			13.9			-			-
	Blanched groundnut	AFB1		1	7.7		-	-		2	7.0–33.4
		AFT			9.0			-			8.2–37.7
	Organic groundnut kernel	AFB1		8	9.2–100		-	-		-	-
		AFT			9.2–120			-			-
Ethiopia	Ground berbere (mixed spices)	AFB1	4	1	-	1	1	15.8	1	1	5.3
		AFT			-			50.4			18.0
		OTA			28.21			-			-
	Chilli powder	AFB1		2	13.5		-	-		-	-
		AFT			15.4–40.8			-			-
	Red pepper powder	AFB1		1	7.63		-	-		-	-
		AFT			18.34			-			-
Gambia	Groundnut	AFB1	22	5	38.3–451	-	-	-		-	-
	Groundnut kernel	AFB1		10	30.8–790		-	-		-	-
		AFT			59.0–790		-	-			-
	Groundnut for feed	AFB1		3	90.5–482		-	-		-	-
	Groundnut kernel for feed	AFB1		4	77.1–210		-	-		-	-
Ghana	Melon seed	AFB1	-	-	-	-	-	-	3	1	5.0
		AFT			-			-			5.4
	Suya pepper	AFB1		-	-		-	-		1	299.0
		AFT			-			-			356.2
	Banku mix	AFB1		-	-		-	-		1	106.0
		AFT			-			-			119.0
Madagascar	Groundnut	AFB1	1	1	99.0	-	-	-	-	-	-
		AFT			135			-			-
Mali	Groundnut paste	AFB1	3	3	203–250	-	-	-	-	-	-
		AFT			297–371			-			-
Morocco	Bitter almond	AFB1	1	1	12.0	-	-	-	-	-	-
		AFT			79.0			-			-
Nigeria	Groundnut	AFB1	2	1	13.0	1	1	437	6	2	>48–50.4
		AFT			15.8			-			>60–76.0
	Groundnut kernel	AFB1			-			-		1	5.3
		AFT			-			-			6.6
	Kuli-kuli spice	AFB1			-			-		1	23.1
		AFT			-			-			27.3
	Dry roasted cocktail groundnut	AFB1			-			-		1	8.6
		AFT			-			-			10.0
	Organic roasted groundnut	AFB1			-			-		1	9.1
		AFT						-			13.7
	Spice mixture	AFB1		1	110			-		-	-
		AFT			154			-		-	-
Senegal	Groundnut	AFB1	5	2	18.3–119	5	-	-	1	-	-
		AFT			20.9–119						
	Groundnuts paste	AFB1		-	-		2	23–104		-	-
		AFT			-			34–140			
	Groundnut kernel	AFB1		1	76		1	43		-	-
		AFT			84			87			
	Groundnuts kernel for bird feed	AFB1		1	43.8		1	33.8		-	-
	Groundnuts powder	AFB1		1	72		1	47		-	-
		AFT			83			81			-
	Peanut butter	AFB1		-	-		-	-		1	8.3
		AFT			-			-			9.2
Sudan	Groundnut	AFB1	12	7	2.8–200	6	2	7.3–781	-	-	-
		AFT			2.8–200			8.1–975			-
	Groundnut kernel	AFB1		3	140–260		4	41–430		-	-
		AFT			160–260			47–430			-
	Whole groundnut	AFB1		1	144		-	-		-	-
		AFT			144			-			-
	Groundnut for bird feed	AFB1		1	170		-	-		-	-
		AFT			170			-			-
South Africa	Groundnut kernel	AFB1	1	1	59.9	-	-	-	-	-	-
Tanzania	Groundnut	AFB1	1	1	53.3	-	-	-	-	-	-
Togo	Groundnut	AFB1	1	1	163	-	-	-	-	-	-
		AFT			177		-	-		-	-
Zambia	Groundnuts for birdfeed	AFB1	3	2	30.1–104	-	-	-	-	-	-
	Groundnut kernels for birdfeed	AFB1		1	59.9		-	-		-	-

^a^No./year—number of European border rejections per year, ^b^No./product/year—number of products rejected per year, conc.—concentration, AFB1—aflatoxin B1, AFT—total aflatoxins, OTA—ochratoxin A.

## Data Availability

Not applicable.
